# Selective androgen receptor modulator use and related adverse events including drug-induced liver injury: Analysis of suspected cases

**DOI:** 10.1007/s00228-023-03592-3

**Published:** 2023-12-07

**Authors:** Natalia Leciejewska, Karol Jędrejko, Víctor M. Gómez-Renaud, Josué Manríquez-Núñez, Bożena Muszyńska, Andrzej Pokrywka

**Affiliations:** 1 Department of Animal Physiology, Biochemistry and Biostructure, Poznań University of Life Sciences, 60-637 Poznan, Poland; 2 Department of Pharmaceutical Botany, Faculty of Pharmacy, Jagiellonian University Medical College, Medyczna 9 Street, 30-688 Kraków, Poland; 3 Human Performance Laboratory, School of Physical Education, Autonomous University of Nuevo Leon, San Nicolas de los Garza, Mexico; 4 Department of Research and Graduate Studies in Food Sciences, School of Chemistry, Autonomous University of Queretaro, Santiago de Queretaro, Mexico; 5 Department of Biochemistry and Pharmacogenomics, Faculty of Pharmacy, Medical University of Warsaw, Warsaw, Poland

**Keywords:** Selective androgen receptor modulators, Unauthorized ingredients, Unapproved pharmaceuticals, Dietary supplements, Safety, Adverse events, Liver injury

## Abstract

**Purpose:**

Selective androgen receptor modulators (SARMs) have demonstrated agonist activity on the androgen receptor in various tissues, stimulating muscle mass growth and improving bone reconstruction. Despite being in clinical trials, none has been approved by the Food and Drug Administration (FDA) or European Medicines Agency for pharmacotherapy. Still, SARMs are very popular as performance-enhancing drugs. The FDA has issued warnings about the health risks associated with SARMs, but the long-term exposure and possible adverse events still need to be fully understood. This review aims to evaluate the adverse events associated with using SARMs by humans.

**Methods:**

PubMed database was searched from September 16, 2022, to October 2, 2023. In total, 20 records were included in the final review. Data from preclinical and clinical studies supported the review.

**Results:**

Since 2020, 20 reports of adverse events, most described as drug-induced liver injury associated with the use of SARM agonists, have been published. The main symptoms mentioned were cholestatic or hepatocellular liver injury and jaundice. Limited data are related to the dosages and purity of SARM supplements.

**Conclusion:**

Promoting SARMs as an anabolic agent in combination with other performance-enhancing drugs poses a risk to users not only due to doping controls but also to health safety. The lack of quality control of consumed supplements makes it very difficult to assess the direct impact of SARMs on the liver and their potential hepatotoxic effects. Therefore, more detailed analyses are needed to determine the safety of using SARMs.

## Introduction

Selective androgen receptor modulators (SARMs) are a group of compounds with therapeutic potential. SARMs act as ligands by diffusing into the cell and binding to the androgen receptor in the cytoplasm. This creates a receptor–ligand complex that translocate to the nucleus where it binds to DNA and acts as a transcriptional regulator of androgen genes response. Unlike natural ligands of this receptor, SARMs have a tissue-selective effect, which gives them a significant advantage over other steroidal anabolic substances [1]. Currently, only SARMs antagonists, such as flutamide, nilutamide, bicalutamide, and enzalutamide, have been introduced to pharmacotherapy as nonsteroidal antiandrogen drugs for the treatment of prostate cancer. However, SARM agonists, which have shown the potential to stimulate muscle growth (anabolic effect) and improve bone reconstruction, are undergoing clinical trials and have not yet been approved by the Food and Drug Administration (FDA) or European Medicine Agency (EMA) for pharmacotherapy [2].

For the first time, a method for detecting SARM agonists (arylpropionamide derivatives) was proposed in spiked urine specimens using liquid chromatography/electrospray ionization tandem mass spectrometry with monitoring and simultaneous precursor ion scanning. The primary reason for developing this assay was to detect the potential misuse of SARMs as a doping agent by elite athletes. Since 2008, SARMs have been included on the World Anti-Doping Agency (WADA) Prohibited List in the class of anabolic agents [3, 4]. Currently, SARMs are still recognized as doping agents and covered by the WADA Prohibited List in the group S1 Anabolic Agents (Other anabolic agents in subsection 2) [5]. In 2009, SARMs were first detected in products available on the market [6, 7]. Despite this, SARM agonists are still available for sale. Some products are labeled as dietary supplements, while others do not have a specific classification or contain statements such as “Not for human consumption” or “Research use only.” SARMs available for sale online are offered in the form of tablets/capsules, liquid, or powder [8–10].

Only a few studies have reported on the prevalence of SARM use in recreational exercisers. In a study conducted in Greece, among 170 adolescent gym users surveyed using a questionnaire, 9% reported using products containing anabolic–androgenic steroids, prohormones, SARMs (including LGD-4033 and MK-2866), and aromatase inhibitors [11], [12].

A more precise estimate of the prevalence of SARMs use comes from a cross-sectional study conducted in the Netherlands. In this study, a completed online questionnaire was used to collect data from a group of young male gym users (*n* = 2269; aged 24 ± 6 years). The study found that 2.7% of all participants reported using SARMs [13]. The most commonly used SARMs were ligandrol (LGD-4033), enobosarm (MK-2866), also known as ostarine, and testolone (RAD-140). The majority of recreational SARMs users are males aged 18–29 years, who consume the substances individually or in stacks. Furthermore, these users have reported various adverse events (AEs) after 3 months of use, including but not limited to mood swings, decreased testicular size, and acne [14].

In 2012, data from WADA adverse analytical findings (AAF) reported only five AAF related to SARMs. However, the number of AAF increased in the following years, reaching its peak in 2019 with ostarine—74, ligandrol—62, RAD-140—4, and single cases of SARM S-23 and andarine. The latest available data from 2020 reported a decrease in these results. However, it should be emphasized that the total number of samples collected was 46.1% lower in 2020 compared to 2019 [5]. SARMs have led to annual increases in positive test results through detection methods in different biological samples, such as hair, nails, urine, and blood [15]. The presence of SARMs in biological samples may be unintentional and unconscious and result from contamination of dietary supplements with microdoses [16].

Recent cases of doping in Olympic and professional sports have involved ostarine and S-23 in athletics and basketball, respectively, as well as LGD-4033 in canoeing. However, the actual prevalence of SARM use is likely to be higher among fitness enthusiasts [17]. Several cases of SARMs detection in athletes have been reported previously [18, 19]. The popularity of SARMs among elite and competitive athletes is fueled by aggressive online marketing that includes many false and unauthorized health claims attributed to SARMs. One frequently used false argument in online advertising is that SARMs are a safe alternative to AAS and do not cause adverse effects. While SARMs do not cause the typical androgenic side effects specific to AAS, the short-term and long-term effects of AAS use and related adverse effects are recognized and expected. In the case of SARMs, the long-term exposure and possible adverse effects are not fully known, which confirms that no molecules from the SARM agonist group have been approved for pharmacotherapy [13, 20, 21].

The FDA has issued a warning letter about the health risks associated with the use of body-building products containing SARMs, informing about the potential increase in the risk of heart attack or stroke and other life-threatening adverse reactions such as liver damage [22, 23]. A large number of notifications about SARM detection have been registered in the database CFSAN Adverse Event Reporting System (CAERS) as potential AEs (The CFSAN 2022).

Warnings about the presence of unauthorized ingredients from SARMs such as ligandrol, ostarine, and testolone in food supplements sold online have been reported on the RASFF panel, mainly from Poland [24].

Most of the AEs associated with the intake of SARMs are drug-induced liver injuries (DILI). DILI can be divided into two groups: intrinsic and idiosyncratic. The intrinsic type includes drugs that produce DILI in a dose-related manner with a predictable capacity, and the rate of occurrence is high when the drug is given in high doses, such as acetaminophen (paracetamol) or selected plant raw materials containing pyrrolizidine alkaloids [25].

Most DILI cases are classified as idiosyncratic, where the drug reaction is unpredictable and not related to the known pharmacological action of the drug, and the rate of occurrence is low. This category includes drugs such as isoniazid, selected antibiotics, statins, and selected ingredients in dietary supplements. An immune response is important in the pathogenesis of idiosyncratic DILI. The threshold of serum alanine aminotransferase (ALT), alkaline phosphatase (ALP), aspartate aminotransferase (AST), and total bilirubin (TB) is used to assess severity of DILI. DILI can be classified as hepatocellular (predominantly an elevation of ALT), cholestatic (mostly elevated ALP), and mixed type of liver damage when the elevation of ALT and ALP is between them. DILI severity is evaluated according to Hy’s Law. Hy’s law assessments are used by the FDA in drug development, including serum activity of ALT of at least three times the upper limit of normal (ULN), and ALT > 3 ULN and TB > 2 ULN without a significant ALP (< 2 × ULN) increase. The “New Hy’s Law” proposed by the Spanish DILI Registry includes a specific factor signed as “nR” and calculated based on dependencies, where (ALT or AST whichever higher/ULN)/(ALP/ULN), and if the result is > 5 and TB > 2 ULN, then nR is considered positive, regardless of the ALP value. To identify and classify DILI cases, the values of aminotransferases, ALP, and TB are used, and serum ALT has greater liver tissue specificity than serum AST [26]. Information resources about DILI and specific drugs that induce them are included in the LiverTox database, but there is no information about SARM agonists [27].

Recently, there have been many reports of liver damage caused by SARMs, as well as comprehensive reviews of the probable causative mechanisms [28, 29]. Our review also takes into account changes in carbohydrate and lipid metabolism, including studies on animal models. Moreover, we summarized the toxicophores in more common SARMs on the black market. Emerging work indicates a problem with the potential risk posed by the use of SARMs and a comprehensive analysis is necessary to better understand the causes of toxic effects.

The aim of this review is to evaluate liver injury cases associated with the use of SARM agonists by humans and to assess their safety according to the most current available knowledge.

## Methodology

To collect data, we searched PubMed for articles published from September 16, 2022, to October 2, 2023, using the search strategy: “((selective androgenic receptor modulators) OR (SARM)) AND ((safety) OR (health risk) OR (adverse event) OR (adverse reaction) OR (side effect) OR (hepatotoxicity) OR (liver injury) OR (drug‐induced liver injury)).”

The first queries provided 341 records, which were screened to exclude 191 records due to their review or systematic review status. Only full-text articles were assessed for eligibility, and in the next stage, all records were screened by title and abstract. We excluded 150 records that were not related to the aim of the review and basic queries, and we added 4 records from other sources.

**Fig. 1:**
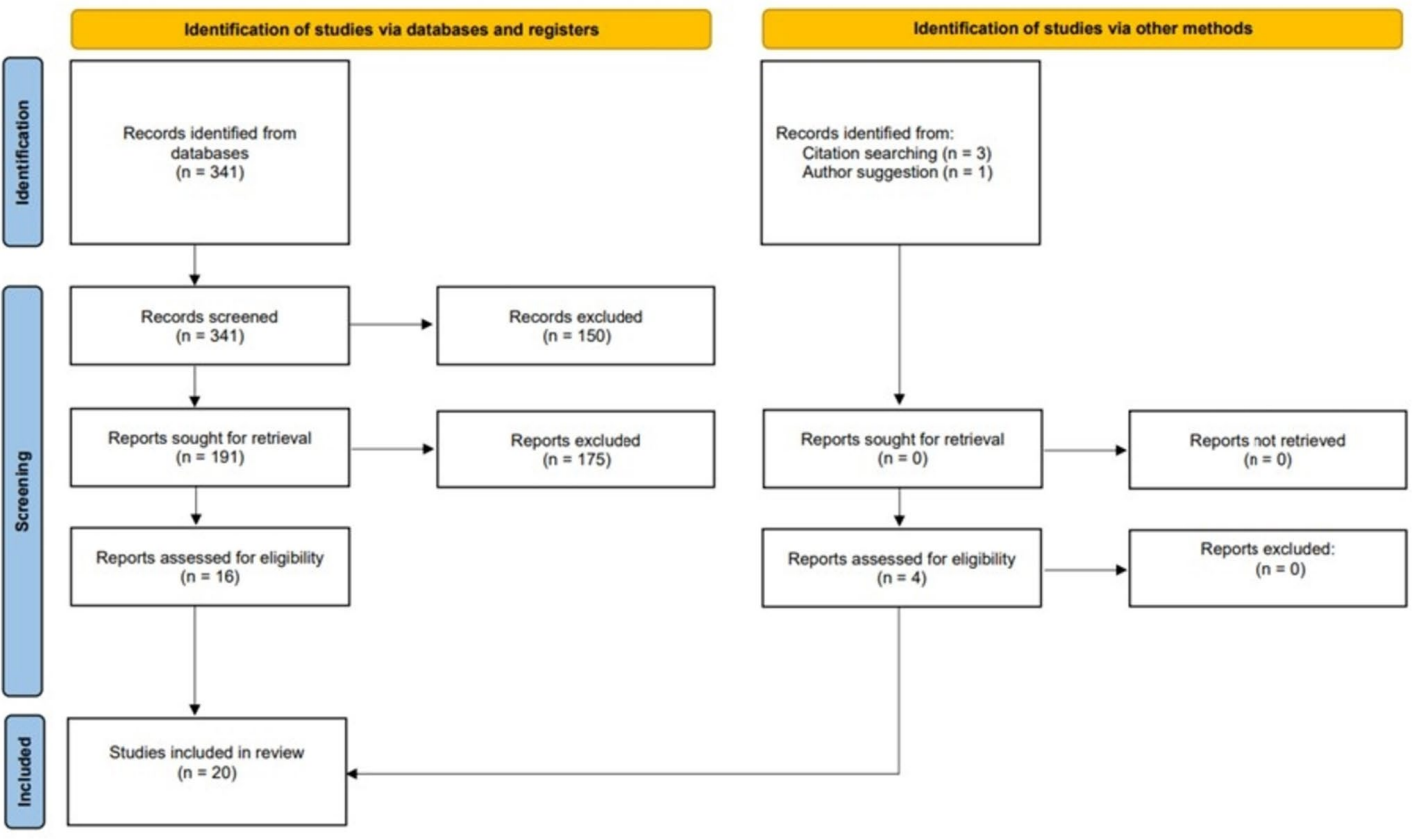
Flowchart outlining the methodology and data workflow

In total, 20 records were included in the final review. The methodology and data workflow is demonstrated in Fig. 1.

## Results

Liver injury has been reported in humans using SARMs, mainly through spontaneous reports. For example, a case of severe DILI with cholestatic hepatitis and perisinusoidal fibrosis was reported in a subject who declared an intake of ligandrol at a dose of 10 mg/day [30]. However, clinical trials have determined that the safe daily doses of ligandrol are 0.1, 0.3, and 1 mg for 21 days. This suggests that the subject was using a much higher dose than what was reported in the clinical trial. Nonetheless, this AE report has some limitations, such as no performed analytical test to detect ligandrol in the suspected product, or no performed toxicological test of blood (or hairs) sample to confirm (or exclude) a share of ligandrol. Details for all collected AEs associated with the use of SARMs agonists are shown in Table 1.

**Table 1:** Assessment of adverse events (mainly liver injury) associated with SARM intake

**Reference**	**Reported substance(s) and dose**	**Primary symptoms**	**Outcome**	**Time of onset**	**Concomitant medication, drugs, or dietary supplements**	**Changes in marker levels**	**Exclusion of other causes (viral hepatitis, autoimmune and metabolic liver diseases)** **Medical history**	**Previous information on hepatotoxicity of the drug**	**Medical history**	**Response to re-exposure** **Comment**
Barbara et al. [30]	Ligandrol (LGD-4033) 10 mg/day	Jaundice, abdominal pain, fatigue, nausea, malnourished	Severe DILI Cholestatic hepatitis and perisinusoidal fibrosis	14 days	No	ALT 229 IU/L; AST 91 IU/L; ALP 425 IU/L; TB 35 mg/dL	Serological markers negative	No	No medical history	Analytical testing for SARMS not performed. This information provided only as per label of product Reported biopsy
Flores et al. [31]	LGD-4033	Jaundice, anorexia, weight loss, nausea, lethargy	Moderate Cholestatic liver injury	9 weeks	No	ALT 273 IU/L; AST 111 IU/L; ALP 289 IU/L; TB 6.8 mg/dL	Negative	No	No medical history	Confirmed detected on ultra-high performance liquid chromatography/photodiode array–mass spectrometry (UHPLC/PDA-MS)
	Testolone (RAD-140)	Jaundice	Moderate Hepatocellular-cholestatic liver injury	4 weeks	Venlafaxine	ALT 54 IU/L; AST 59 IU/L; ALP 327 IU/L; TB 17 mg/dL	Negative	No	Depression	Confirmed detected on UHPLC/PDA-MS
Barbara et al. [32]	LGD-4033 (5 mg) RAD-140 (7.5 mg)	Jaundice, abdominal pain, diarrhea, pruritus	Severe Cholestatic liver injury	7 weeks	Marijuana	ALT 46 IU/L; AST 36 IU/L; ALP 529 IU/L; TB 34.5 mg/dL	Negative	No	History of alcohol use	Analytical testing for SARMS not performed. This information provided only as per label of product Reported biopsy
Baliss et al. [33]	RAD-140	Jaundice, abdominal pain, pruritus	Not mentioned	Not mentioned	No	ALT 293 IU/L; AST 145 IU/L; ALP 122 IU/L; TB 8.4 mg/dL	Negative	No	Asthma	Dose was not specified
Koller et al. [34]	LGD-4033	Jaundice, dark urine	Cholestatic liver injury	3–4 weeks	No	R factor 1.6; ALT 132 IU/L; AST N/A; ALP 92.4 IU/L; TB 13.9 mg/dL	Negative	No	No medical history	Dose was not specified Reported biopsy
	LGD-4033 Ostarine (MK-2866)	Jaundice, nausea, fatigue	Cholestatic liver injury	3–4 weeks	Dietary supplements such as protein, amino acids, fat burners	R factor 2.0; ALT 145 IU/L; AST N/A; ALP 92 IU/L; TB 23.5 mg/dL	Negative	No	No medical history	Dose was not specified Reported biopsy
Bedi et al. [35]	MK-2866	Jaundice, anorexia, diarrhea, weight loss, lethargy	Moderate to severe Cholestatic liver injury	2 months	Finasteride, zopiclone	ALT 112 IU/L; AST 69 IU/L; ALP 268 IU/L; TB 19.9 mg/dL	Negative	No	No medical history	Dose was not specified Reported biopsy
Kintz et al. [36]	Cardarine (GW-1516) MK-2866	Epigastric pain, myalgia pain, headache, brown urine	Rhabdomyolysis Liver cytolysis	MK-2866 1 day; GW-1516 4 days	No	ALT 922 IU/L; AST 2558 IU/L; ALP N/A; TB N/A	N/A	N/A	No medical history	The biological samples (blood, urine, hair) were tested by LC–MS/MS
Lam and Wong [37]	Name of SARMs not specified	Jaundice, fatigue, vomiting	Cholestatic injury	Not mentioned	No	ALT 92 IU/L; AST 48 IU/L; ALP 105 IU/L; TB 12.4 mg/dL	Negative	No	No medical history	Analytical testing for SARMS not performed
Akhtar et al. [38]	RAD-140 LGD-4033 MK-2866	Jaundice, abdominal pain, pruritus	Cholestatic liver injury	24 weeks	No	ALT 115 IU/L; AST 61 IU/L; ALP 173 IU/L (max. peak 434 IU/L); TB 12.4 mg/dL	Negative	Acetaminophen	Average alcohol intake	Dose was not specified
Lee et al. [39]	LGD-4033 RAD-140 YK-11	Jaundice, scleral icterus, decrease appetite, pruritis	Cholestatic liver injury	3 months	No	ALT 148 IU/L; AST 88 IU/L; ALP 151 IU/L; TB 29.2 mg/dL	Negative	No	Average alcohol intake	Dose was not specified
Padappayil et al. [40]	RAD-140	Shortness of breath	Acute myocarditis	Period of intake not specified Reported only that it was used 1 day before hospitalization	Insulin, buprenorphine sublingual	C-Reactive protein 147.22 mg/L; Troponin I 77.11 ng/mL	N/A	No	Type I diabetes mellitus History of cocaine and heroin abuse	Dose was not specified
Weinblatt and Roy [41]	MK-2866	Itching, dark-colored urine	Hepatocellular-cholestatic liver injury	3 weeks	No	ALT 346 IU/L; AST 110 IU/L; ALP 123 IU/L; TB 0.5 mg/dL	Negative	No	Average alcohol intake	Dose was not specified
Khan et al. [42]	LGD-4033	Jaundice, scleral icterus, dark urine, abdominal pain, nausea, vomiting, diarrhea, fatigue, pruritus	Cholestatic liver injury	4 weeks	Pre-workout dietary supplements	AST 79 IU/L; ALT 165 IU/L; ALP 213 IU/L (max. peak 529 IU/L); TB 16.9 mg/dL	Negative	No	Average alcohol intake	Dose was not specified Reported biopsy
Sotorník et al. [43]	RAD-140 5 mg twice daily Andarine 25 mg twice daily	Polydipsia, polyuria, blurred vision, weight loss	Hyperglycemia Hypertension	9 weeks	Growth hormone (GH) secretagogue (GHS)/ghrelin analogue—ibutamoren	Data unavailable	Data unavailable	No	3 years earlier—metabolic syndrome, dyslipidemia, liver steatosis	Insulin therapy
Leung et al. [44]	RAD-140 15 mg daily	Jaundice, abdominal pain, scleral icterus	Cholestatic liver injury	5 weeks	Acetaminophen, aspirin, caffeine	ALT 171 IU/L; AST 71 IU/L; ALP 151 IU/L; TB 10.8 mg/dL ALT 125 IU/L; AST 82 IU/L; TB 32.3 mg/dL	Negative	Acetaminophen	No medical history	Analytical testing for SARMS not performed. Information provided only as per label of product Reported biopsy
Malave [45]	LGD-4033 S-23	No reported	Hepatocellular liver injury	8 weeks	No	AST 75 IU/L; ALT 144 IU/L; ALP 56 IU/L (max. peak 73 IU/L); TB 0.8 mg/dL	Negative	No	No medical history	Not mentioned
Arayangkool et al. [46]	LGD-4033 Ostarine RAD-140	Jaundice Pruritus Weight loss Abdominal pain	Cholestatic liver injury Canalicular bile plugs Hepatocellular dropout Acute tubular injury with pigmented bile casts	8 weeks	No	AST 52 IU/L ALT 52 IU/L ALP 343 IU/L TB 43.3 mg/dL	Negative	No	No medical history	Analytical testing for SARMS not performed. Dose was not specified Reported biopsy
Ladna et al. [47]	RAD-140	Nausea, vomiting, severe right upper quadrant abdominal pain, jaundice, dark urine, constipation	Acute liver injury	8 weeks	No	AST 51 IU/L ALT 243 IU/L TB 4.9 mg/dL	Negative	No	No medical history	Analytical testing for SARMS not performed
Mohamed et al. [48]	RAD-140	Jaundice, nausea, fatigue, dark urine, acholic light gray-colored stool, pruritus	Mixed portal hepatitis, cholestasis, biliary reactive changes	16 weeks	Performance-enhancing supplement	AST 70.8 IU/L (1.18 µkat/L) ALT 99.6 IU/L (1.66 µkat/L) ALP 318 IU/L (5.3 µkat/L) TB 427.5 mg/dL	Negative	No	No medical history	Analytical testing for SARMS not performed. Dose was not specified Reported biopsy
Cardaci et al. [49]	LGD-4033 10 mg daily	Controlled study—not reported	Controlled study—not reported	5 weeks	MK-677 15 mg daily, ibutamoren (GH secretagogue)	AST 47 IU/L ALT 61 IU/L ALP 41 IU/L TB 0.3 mg/dL	Negative	No	No medical history	Reported muscle biopsy

In most cases, increased liver enzymes were identified. Elevated alanine aminotransferase (ALT), aspartate aminotransferase (AST), and lactate dehydrogenase (not measured in the reported cases) are considered to be indicative of cell damage. Alkaline phosphatase (ALP) and total bilirubin (TB) were also measured in reported cases. Generally, hepatocellular damage is indicated by increased aminotransferase activity, while higher ALP and GTTP activity indicates cholestatic liver injury.

Among the reported cases of oral use of SARMs, most often cholestatic liver damage was diagnosed. SARMs are typically used orally and the mechanism of liver damage may be similar to 17α-alkylated AAS [28] and directly contribute to a highly characteristic form of acute cholestasis, ranging from very mild to severe. Patients diagnosed with cholestatic liver injury had characteristic symptoms including nausea, pruritus, fatigue, jaundice, and dark urine and those were the main reasons for being admitted to the hospital.

These disorders occurred regardless of the SARM used—they accompanied the intake of ostarine, RAD-140, and LGD-4033 individually [31], [34] as well as in the combination of these three SARMs [46]. No additional supplementation was reported in any of these cases. Among the remaining reported cases of cholestatic liver injury, patients used combinations of several SARMs [39] as well as in combination with other substances, such as finasteride and zopiclone [35], an unnamed pre-workout supplement [42], and a mix of acetaminophen, caffeine, and aspirin [44].

Other results related to liver damage concerned perisinusoidal fibrosis, where the patient only declared taking LDG-4033 [30]. The remaining results concerned hepatocellular liver injury due to the intake of LGD-4033 and S-23 [45] as well as liver cytolysis due to the combination of ostarine and the metabolism modulator GW-1516 [36].

Only in a few cases was the dose of the substances taken precisely determined, but it should be noted that no laboratory analysis was performed to confirm the purity and content of the substances. Only one study found the content of LGD-4033 and RAD-140 [31], while the second one analyzed biological samples (blood, urine, and hair) obtained from the patient [36].

Occurrences unrelated to liver injury included acute myocarditis. This was the first reported case of a SARM that may have a causal relationship with acute myocarditis. However, the patient had a medical history of type 1 diabetes which was being controlled with insulin injections and was also undergoing opioid-assisted treatment with sublingual buprenorphine due to a history of drug abuse. The case is ambiguous and it is unclear whether acute myocarditis was caused by the SARM or by the additional medications administered to the patient. The report suggests that there may be a potential interaction between SARMs and insulin and/or opioid medications [40]. Arayangkool et al. described the case of a patient who also suffered from bile cast nephropathy because of SARM-associated drug-induced liver injury [46].

## Discussion

To address the potential inconsistencies in spontaneous AE reports, the authors summarized the duration of exposure, dosage, safety assessment, and pharmacokinetic parameters of selected SARM agonists covered in clinical trials. This information is accessed in Supplementary section. It should be noted that preclinical studies on the andarine (S-4) compound, which is also available for sale online, were suspended and did not advance to phase I of clinical trials [2].

In the clinical trials conducted with SARM agonists, some participants experienced elevations in AST/ALT/TB levels. However, these trials used controlled and precise doses of the investigational product. In the case reports of DILI, the exact dose of SARMs used by the subjects was not specified in most cases. In some cases where the dose was mentioned, it was found to be several times higher than the dose used in clinical trials, but this information was based only on the label of the product and not confirmed through analytical testing.

**Fig. 2:**
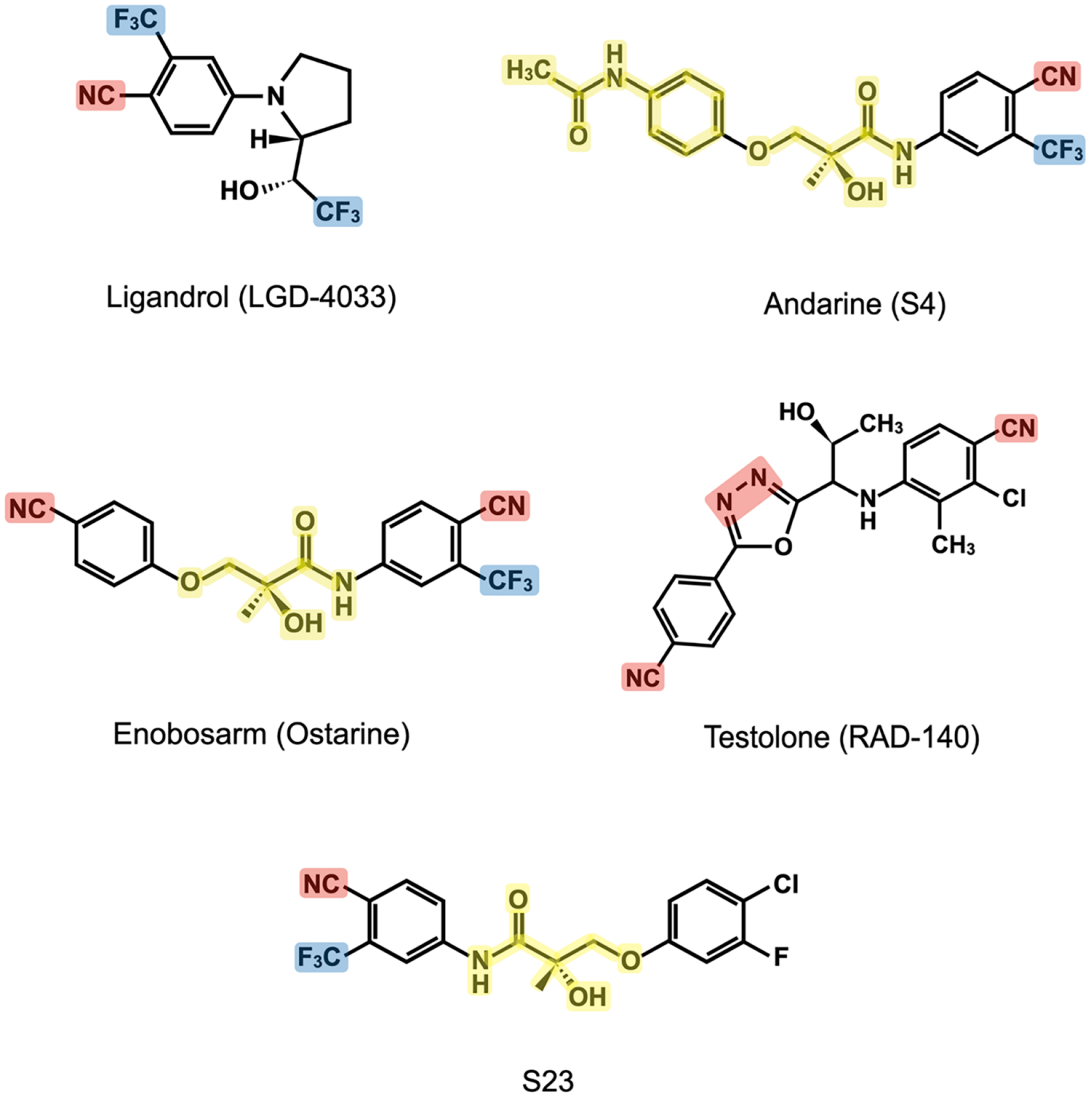
Various toxicophores in SARM molecules. A red highlight represents toxicophores; blue highlight represents detoxicophores; yellow highlight represents potential unstable substructure

It is important to note that the selected chemical structures of SARMs contain various toxicophores, which are well recognized in medicinal chemistry as potential causes of toxicity in drugs (Fig. 2). For example, ligandrol contains a nitrile substituent (Ar–CN) and an aziridine moiety, while testolone contains two Ar–CN groups and an aromatic azo group (Ar–N = N–Ar). Andarine contains an aromatic nitro substituent (Ar–NO_2_) and a potentially unstable substructure of (Ar–NHCO–C(OH)(CH_3_)–CH_2_–O–Ar), which is also found in ostarine, along with two Ar–CN groups. This substructure, which is similar to the main toxicophore of paracetamol (acetaminophen), can be unstable and form reactive and hepatotoxic *N*-acetyl-*p*-benzo imine derivatives during oxidative metabolism in the liver. This is especially concerning in cases where the substance is administered at an unknown or uncontrolled dosage or when multiple substances are taken in a single dose. The presence of selected toxicophores, such as Ar–NO_2_ in andarine, may be associated with higher toxicity of this compound [50–53].

Previous studies have shown that out of 44 dietary supplements sold as SARMs, 39% of them contained unapproved substances other than SARMs, such as ibutamoren (a growth hormone secretagogue), cardarine (GW501516, a peroxisome proliferator-activated receptor-δ agonist), and SR9009 (Rev-Erba [a circadian clock protein] agonist). Mass spectrometry analysis of these dietary supplements revealed that only 52% of them contained SARMs, indicating that many of these products were mislabeled [8–10, 15].

The CAERS database provides interesting observations, including many reports of potential AEs for specific keywords/queries related to SARMs (Table 2). We searched the CAERS database using the following keywords: “SARM,” “andarine,” “S-4,” “ostarin(e),” “MK-2866,” “ligandrol,” “LGD-4033,” “testolone,” “RAD-140,” and “YK-11.” In summary, the reports not only included cases of liver injury but also blindness or a visual impairment, cerebrovascular accidents, paresthesia, abnormal hormone levels, testicular disorders, gynecomastia, increased blood prolactin, sexual dysfunction, altered mood, and a single fatal case of cardiac death [54].

**Table 2:** CAERS number of reports in CAERS for a specified keyword and alternative queries

**Selected keyword**	**Number of reports**	**Product**	**Year of report**	**Subject**	**MedDRA “Preferred Terms”**	**Outcome**
“SARM”	15	DNA ANABOLICS SARM MK 677	12.4.2015	Age not reported, male	Decreased activity, mood altered	Other outcome
		SARM-SELECTIVE ANDROGEN RECEPTOR MODULATORS	9.2.2016	19 years, male	Epinephrine increased, psychotic disorder	Life threatening, visited emergency room
		SARM	7.6.2017	40 years, male	Drug-induced liver injury	Hospitalization, visited emergency room
		SARMS 140	12.27.2018	28 years, male	Drug-induced liver injury, jaundice	Life threatening, hospitalization
		DNA PHARMA SARM IBUTAMOREN MK-677	10.28.2019	30 years, male	Abdominal discomfort, diarrhea, headache, weight decreased	Other outcome
		MAX HEALTH & NUTRITION SARMS	11.12.2019	Age and sex not reported	Blindness	Hospitalization, other serious or important medical event
		BEAST SARMS	2.24.2020	18 years, male	Testicular disorder	Disability
		SARM SP RESEARCH PRODUCT MASS RESEARCH	8.17.2020	47 years, male	Anxiety, blood pressure increased, cerebrovascular accident, insomnia, tachycardia	Hospitalization, other serious or important medical event, visited emergency room
		SAVAGE SARM STACK	9.28.2020	31.8 years, male	Delusion, paranoia	Hospitalization
		PRIME NUTRITION LGXNDS SARMS DESTROYER	3.25.2022	31 years, male	Hormone level abnormal, mood altered	Other serious or important medical event
“andarine” “S-4”	3	S4 (ANDARINE)	6.12.2017	27 years, male	Dizziness, presyncope	Other serious or important medical event
		VIRILITECH ANDARINE	2.22.2018	26 years, male	Acute hepatic failure	Hospitalization, visited a health care provider
		MMG LABS ANDARINE S-4	11.27.2019	Age and sex not reported	Cardiac death	Death
“ostarine” “ostarin” “MK-2866”	6	OSTARINE MK-2866	7.17.2017	36 years, male	Cerebrovascular accident	Hospitalization
		VIRILITECH OSTARINE	2.22.2018	26 years male	Acute hepatic failure	Hospitalization, visited a health care provider
		SARM OSTARIN	9.26.2018	Age not reported, male	Hormone level abnormal	Other serious outcome
		HARDCORE OSTARIN SARM SERIES	2.24.2020	Aged not reported, male	Gynecomastia	Other serious or important medical event
		KN NUTRITION CARDARINE OSTARINE	4.5.2021	30 years, male	Malaise	Hospitalization, disability
		CHEMYO MK-2866 OSTARINE 25MG/ML	11.10.2021	17 years, male	Blood prolactin increased, sexual dysfunction	Disability, visited a health care provider, other serious outcome
“ligandrol” “LGD-4033” “LGD4033” “LGD 4033” “LGD”	6	VIRILITECH LIGANDROL	2.22.2018	26 years male	Acute hepatic failure	Hospitalization, visited a health care provider
		LIGANDROL LGD-4033	5.16.2019	23 years, male	Hepatic failure	Life threatening, hospitalization, disability
		LGD4033	11.21.2019	35 years, male	Congenital anomaly	Congenital anomaly
		LGD 4033	12.17.2020	37 years, male	Cerebrovascular accident, paresthesia	Hospitalization, visited emergency room
		PRIMEVAL LAB SUPER LGD	3.31.2016	Age not reported, male	Mood altered	Other outcome
		CONTINUUM LABS LGD EXTREME	7.6.2016	Aged not reported, male	Bone pain, chest pain, renal disorder, visual impairment	Other outcome
“testolone” “RAD-140” “RAD140” “RAD 140”	0 1 0 2	HARDCORE RAD-140 SARM SERIES	2.24.2020	Aged not reported, male	Gynecomastia	Other serious or important medical event
		SARMS Rad 140	12.27.2018	28 years, male	Drug-induced liver injury, jaundice	Life threatening, hospitalization
		RAD 140	12.17.2020	37 years, male	Cerebrovascular accident, paresthesia	Hospitalization, visited emergency room
“YK-11” “YK11” “YK 11”	2 0 0	YK-11	5.16.2019	23 years, male	Hepatic failure	Life threatening, hospitalization, disability
		YK-11	12.17.2020	37 years, male	Cerebrovascular accident, paresthesia	Hospitalization, visited emergency room

The widespread availability of SARMS can be demonstrated by looking at the additional sources such as the RASFF (summary of findings are included in Supplementary section), at the Polish market (Allegro.pl) as an example, and in the NIH Label Database. All data from the analysis of Polish market and NIH Label Database is presented in Table 3. However, all the information which we analyzed are based only on the description included on the label of the product provided by the producer.

**Table 3:** SARM products available on the market—data from the Poland market (allegro.pl) and the NIH Label Database

**Selected keyword**	**Poland market (Allegro.pl)**	**NIH Label Database**
“SARM”	61	25
“andarine”	8	10
“ostarine” “MK-2866”	55 20	20 6
“ligandrol” “LGD-4033”	14 18	17 3
“testolone” “RAD-140”	1 55	2 5
“YK-11”	19	6

The WADA provides more precise data on antidoping testing figures, which detect all prohibited substances through analytical tests (Table 4) [55].

**Table 4:** SARM detection included in the WADA Anti-Doping Testing Figs. 2011–2020

**SARM molecule**	**Year 2020/occurrence**	**Year 2019/occurrence**	**Year 2018/occurrence**	**Year 2017/occurrence**	**Year 2016/occurrence**	**Year 2015/occurrence**	**Year 2014/occurrence**	**Year 2013/occurrence**	**Year 2012/occurrence**	**Year 2011/occurrence**
Enobosarm (ostarine)	37	74	45	47	28	28	15 results for all SARMs	13 results for all SARMs	5 results for all SARMs	1 result for all SARMs
LGD-4033 (ligandrol)	29	62	26	9	6	2				
RAD-140 (testolone)	4	4	5	6	2	–				
S-4 (andarine)	1	1	1	3	1	2				
S-23	–	1	–	–	–	–				
S-22	–	–	–	–	2	–				

– Absent

At first, the heterogeneity of SARMs’ chemical structures posed a challenge to the development of precise detection methods. However, in subsequent years, there has been intensive development of new testing assays [6, 7].

Sobolevsky et al. provided the first human excretion results on ligandrol and confirmed several hydroxylated metabolites, including monohydroxylated and bishydroxylated, as well as hydroxylated and ring-cleaved metabolites [56].

Some potential mechanisms underlying liver damage from the use of SARMs as performance-enhancing substances have not been well studied [57]. In reported cases, increased liver enzymes indicating cell damage as well as cardiac muscle damage were reported. The recently published report on the profibrotic and cardiotoxic effects of ostarine may indirectly indicate the direction of further research [58]. One possible explanation is that SARMs significantly increase carbohydrate metabolism, particularly gluconeogenesis, resulting in hyperglycemia and insulin resistance [59]. The rate of gluconeogenesis in the liver is largely regulated by the activity of FOXO1 and PGC-1α [60], although their exact relationship in the context of SARM induction remains unclear.

Interestingly, PGC-1α is also involved in fatty acid metabolism, specifically increasing beta-oxidation in the liver and playing a crucial role in metabolic adaptation during starvation in this tissue [61]. The overstimulation of both pathways due to SARM-induced anabolism can lead to oxidative stress and insulin resistance in hepatocytes [62], which may in turn increase proinflammatory mechanisms such as interleukin secretion [63] and peroxidated molecule production, further contributing to the inflammatory cascade [64]. Ultimately, these processes may activate apoptotic cascades [65].

The effect of SARMs on lipid metabolism is an area that requires further investigation, as the available literature on the anabolic effect of SARMs poorly reports their effects on nontarget tissues and the liver. However, changes in lipid metabolism have been reported in animal models, including in the liver [66, 67], plasma [68, 69], and adipose tissue [69, 70]. Table 5 summarizes the reported changes in lipid metabolism in animal models. Excess body fat can have adverse effects on metabolic changes due to the adipokines it produces. Ostarine has been shown to reduce the secretion of leptin and adiponectin from white adipocytes [71], while low levels of leptin can intensify de novo lipogenesis in the liver and promote lipid accumulation in muscles, affecting insulin production in the pancreas and contribute to insulin resistance [72–74]. Similarly, low levels of adiponectin can promote negative effects such as oxidative stress and mitochondrial dysfunction in the liver [75, 76]. The results of Min et al. using another SARM, S-42, reported no change in the level of adiponectin [67]. In addition, the results show the downregulation of SREBP-1c factors as well as FAS, which are crucial elements in lipogenesis de novo. This is the opposite of the results obtained using SAA [77].

**Table 5:** Summary of selected changes in lipid metabolism after SARM treatment in an animal model

**Metabolic changes**	**Model**	**SARM molecule**	**Reference**
**Liver**			
Weight↑	ORX rats	Ostarine	Komrakova et al. [66]
SREBP-1c mRNA expression↓	ORX rats	S-42	Min et al. [67]
FAS mRNA expression↓	ORX rats	S-42	Min et al. [67]
**Blood serum**			
TG↓	Monkeys Female rats OVX	SARM-2f MK-0773	Morimoto et al. [68] Schmidt et al. [69]
Total cholesterol↑	Male rats	Ostarine	Komrakova et al. [78]
Total cholesterol↓	Monkeys	SARM-2f	Morimoto et al. [68]
LDL↑	Male rats	LGD-4033 (+ physical activity)	Komrakova et al. [78]
LDL↓	Monkeys Female rats OVX	SARM-2f MK-0773	Morimoto et al. [68] Schmidt et al. [69]
HDL↑	Male rats	Ostarine (+ physical activity)	Komrakova et al. [78]
HDL↓	Monkeys	SARM-2f	Morimoto et al. [68]
**Adipose tissue**			
Fat mass↓	OVX rats OVX rats	S-4 S-4	Kearbey et al. [79] Kearbey et al. [70]
Lipolysis↑	ORX rats Adipocytes from rats	S-42 Ostarine	Min et al. [67] Leciejewska et al. [71]
Lipogenesis↓	ORX rats Adipocytes from rats	S-42 Ostarine	Min et al. [67] Leciejewska et al. [71]
Adiponectin↓ Adiponectin –	Adipocytes from rats ORX rats	Ostarine S-42	Leciejewska et al. [71] Min et al. [67]
Leptin↓	Adipocytes from rats	Ostarine	Leciejewska et al. [71]

*ORX/OVX* orchidectomy/ovariectomy rat osteoporosis model, *LDL* low-density lipoprotein, *HDL* high-density lipoprotein, *TG* triglyceride

Some studies have found changes in lipoproteins, triglycerides, and cholesterol in clinical trials and animal models. Although inconsistent results were noted for triglycerides and low-density lipoprotein in human subjects studies, the lowering of high-density lipoprotein (HDL) confirmed in clinical trials deserves attention [80–82]. Other studies have also reported a reduction in apolipoprotein AI, a main protein in HDL [83–85]. Although these changes may be significant for the cardiovascular system, long-term reduced production of HDL may be related to liver dysfunction and limited regenerative processes of this organ [86, 87]. It is important to note that the results returned to baseline levels after the end of treatment but only concerned a relatively short period of administration (from 14 to 86 days). The longest administration time (113 days and 6 months) did not assess these parameters. The effect of long-term use of SARMs at high doses remains unclear. Summary of selected clinical trials demonstrated in supplementary data [88–91].

The use of SARMs can disrupt metabolic pathways and potentially impact liver metabolism. However, the varying effects observed in studies can be attributed to factors such as the type of SARM, dose, and physiological state of the subjects. Although animal studies may not be entirely reliable due to differences in metabolism, they can provide partial insights into the mechanisms underlying DILI. Given the limited use of SARMs in human studies, animal models with humanized livers, chimeric mice with humanized cytochrome P450 enzymes, or cell models are crucial in identifying the mechanisms involved in DILI.

## Conclusions

Our review provides a comprehensive overview of the harmful effects of SARMs on the liver. However, current knowledge of the toxicity mechanisms of SARMs is insufficient. Uncontrolled dosing and/or combining several SARM compounds in one product may lead to AEs related to liver damage and affect lipid metabolism disorders. Withdrawal of the substance often results in liver recovery, but the actual number of SARM users remains unclear. Analytical tests have confirmed many discrepancies in both quantity and quality analysis, indicating a very low quality of SARM products available on the market. Labels often do not provide accurate information for consumers, and cases of counterfeit and fake manipulation among ingredients and declared doses have been confirmed.

Assessing liver damage, severity, and potential hepatotoxicity of SARM compounds, as well as their causality, can be helpful in the diagnosis and implementation of effective treatment in clinical practice. Promoting SARMs as a safe alternative to other anabolic compounds is significantly dangerous and poses a risk to public health. Increasing consumer awareness of the risks of SARM supplementation is crucial in preventing harmful effects.

## Data Availability

The data that support the findings of this study are available on request.
